# Hybrid Interfaces of 2D Materials with Polymers for Emerging Electronics and Energy Devices

**DOI:** 10.3390/ma19030602

**Published:** 2026-02-04

**Authors:** Jaehyuk Go, Jaehyun Kim, Sanghyeok Ju, Daekyoung Yang, Seongchan Kang, Heekyeong Park

**Affiliations:** School of Electronics Engineering, Kyonggi University, Suwon-si 16227, Gyeonggi-do, Republic of Korea; gjh0407@kyonggi.ac.kr (J.G.); kjh523164@kyonggi.ac.kr (J.K.); wntkd12031@naver.com (S.J.); jm918703@kyonggi.ac.kr (D.Y.); galaxy020811@naver.com (S.K.)

**Keywords:** 2D materials, polymer hybrid, flexible electronics, optoelectronics, memory devices, energy devices

## Abstract

Two-dimensional (2D) materials offer exceptional electrical, optical, and mechanical properties but face challenges in terms of scalability, stability, and integration. Hybridizing 2D materials with polymers provides an effective route to overcome these limitations by enabling tunable interfaces, mechanical compliance, chemical functionality, and three-dimensional device processability. This review summarizes the fundamental structural configurations of 2D–polymer hybrids, including embedded composites, stacked heterostructures, covalently functionalized interfaces, polymer-encapsulated layers, and fiber–network architecture, and describes how their interfacial interactions dictate charge transport, environmental robustness, and mechanical behavior. We also highlight major fabrication strategies, such as solution dispersion, in situ polymerization, and vapor-phase deposition. Finally, we discuss emerging applications in sensors, optoelectronics, neuromorphic systems, and energy devices, demonstrating how synergistic coupling between 2D materials and functional polymers enables enhanced sensitivity, programmable electronic states, broadband photodetection, and improved electrochemical performance. These insights provide design guidelines for future multifunctional and scalable 2D–polymer hybrid platforms.

## 1. Introduction

Nanotechnology has revolutionized materials engineering, enabling the atomic-scale design of functional systems and leading to the emergence of two-dimensional (2D) materials as key platforms for future electronics [1,2,3,4]. Owing to their atomic thickness and unique electronic structures, 2D materials such as graphene, transition metal dichalcogenides (TMDCs), hexagonal boron nitride (h-BN), and MXenes exhibit exceptional electrical, optical, and mechanical properties distinct from bulk counterparts [5,6,7,8]. These materials have enabled multifunctional platforms with enhanced charge transport, tunable bandgaps, great optical response, and mechanical robustness, leading to diverse applications in electronic [9,10,11], optoelectronic [4,12,13], sensors [14,15,16], and energy devices [17,18,19].

Despite these advantages, the practical implementation of 2D materials remains hindered by challenges associated with large-area synthesis, substrate compatibility, and environmental stability. In particular, 2D materials alone often fail to satisfy the demanding requirements of advanced electronic systems, such as chemical selectivity, long-term operational stability, and compatibility with low-temperature fabrication processes. To address these limitations, the hybrid integration of 2D materials with functional counterparts has emerged as a promising strategy.

Among various candidates for hybrid integration, organic polymers offer unique advantages such as solution processability, mechanical compliance, low-temperature fabrication compatibility, and chemical tunability [20,21,22]. Their molecular versatility enables precise control of interfacial, electrical, and chemical properties across diverse applications. In chemical and optical sensors, functional polymers can impart chemical selectivity or enhance light–matter interactions at the 2D interface [23,24,25]. In memory devices, ferroelectric polymers, such as P(VDF-TrFE), facilitate charge trapping and polarization-based switching [26,27], while in energy systems, ion-conducting and redox-active polymers serve as solid or gel electrolytes to improve ionic transport and mechanical stability [28,29]. Additionally, insulating polymers such as PMMA and Su-8 provide surface passivation, dielectric modulation, and environmental protection, collectively expanding the functionality and reliability of 2D material-based hybrid devices [30,31]. Furthermore, 2D-structured polymers and TMD heterostructure exhibit strong interlayer electronic coupling and ultrafast energy transfer at the vdW interface [32].

Therefore, this review aims to present a comprehensive overview of the latest trends in the research of hybrid structures based on 2D materials and polymers. We emphasize the importance of the synergistic effects and interfacial engineering achieved through this hybrid approach. We first highlight the structural configurations and interfacial interactions that define 2D–polymer hybrids, encompassing embedded nanosheet composites, stacked or layered heterostructures, covalently functionalized interfaces, polymer-encapsulated 2D channels, and fiber or network-based hybrid architectures. This structure-oriented perspective enables a coherent interpretation of hybrid systems across different classes of 2D materials and polymers. Furthermore, we survey major fabrication strategies, including solution dispersion, in situ chemical polymerization, and vapor-phase deposition, which enable controlled structural and electronic interactions. Finally, we discuss emerging applications in sensors, optoelectronic and neuromorphic devices, and energy systems, emphasizing how synergistic integration between 2D materials and polymers leads to enhanced performance, multifunctionality, and scalable device engineering. By linking hybrid architecture and interfacial characteristics to application-specific device requirements, we provide a structure-to-function roadmap that supports rational material selection and device design. This review aims to provide a unified framework and design guidelines for researchers seeking to advance next-generation flexible and hybrid electronic platforms through 2D–polymer integration.

## 2. Structural Configurations and Interfacial Interactions of 2D–Polymer Hybrid Structures

**Figure 1:**
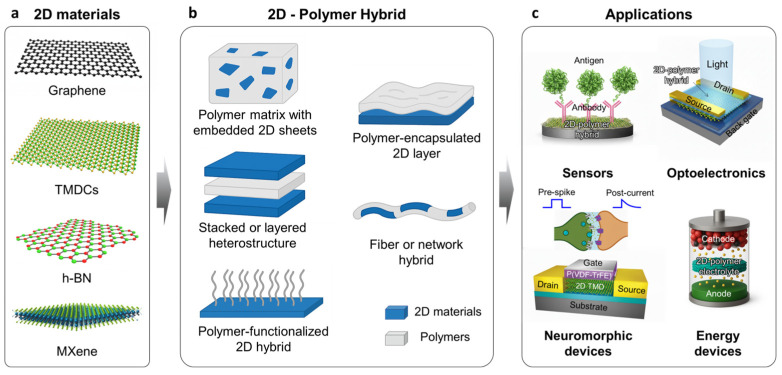
Schematic overview of representative structures and device applications of 2D polymer hybrid systems. (**a**) Four major classes of 2D materials (graphene, TMDCs, h-BN, and MXenes). (**b**) Representative 2D–polymer hybrid structures (polymer matrices embedded with 2D sheets, stacked or layered heterostructures, polymer-functionalized 2D hybrids, and fiber or network architectures). (**c**) Device applications (bio–chemical sensors, optoelectronics, neuromorphic devices, and energy storage devices).

The most widely used 2D materials, including graphene, TMDCs, h-BN, and MXenes, (Figure 1a) exhibit exceptional electrical, optical, and chemical properties, making them promising candidates for various device applications. Hybridizing these 2D materials with polymers provides greater structural tunability for broader application versatility than that which cannot be achieved by 2D materials alone [33]. The structural configuration of a 2D material and polymer hybrid determines how the two components interact and, consequently, how the resulting device performs. Depending on spatial arrangement, interface chemistry, and bonding characteristics, the hybrid can adopt several representative architectures, including polymer matrices with embedded 2D sheets, stacked architectures, polymer-functionalized 2D hybrids, polymer-encapsulated structures, and fiber or network hybrids (Figure 1b). Each configuration provides distinct advantages in terms of charge-transport pathways, mechanical flexibility, interfacial coupling, barrier properties, and environmental durability, thereby enabling the integration of 2D materials into a wide spectrum of device platforms, such as biosensors, optoelectronics, memories, and electrochemical energy storage systems (Figure 1c). Before discussing each hybrid architecture, Table 1 provides a comparative summary of representative 2D–polymer hybrid configurations, their dominant interfacial interaction mechanisms, and the resulting impacts on charge transport, mechanical stability, and device performance, serving as a structural framework for the discussions in this section.

### 2.1. Polymer Matrix with Embedded 2D Sheets

In this configuration, 2D nanosheets are dispersed within a polymer host matrix, forming a continuous composite film in which the polymer provides mechanical integrity and flexibility while the embedded 2D sheets establish electronically or optically active sites that locally modulate charge-transport behavior [28,34,35,36,37]. The interfacial interaction in such embedded systems is generally weak to moderate, dominated by van der Waals forces, hydrogen bonding, and, in some cases, electrostatic or π–π interactions, depending on the surface chemistry of the 2D material and the polymer [38,39,40,41].

**Figure 2:**
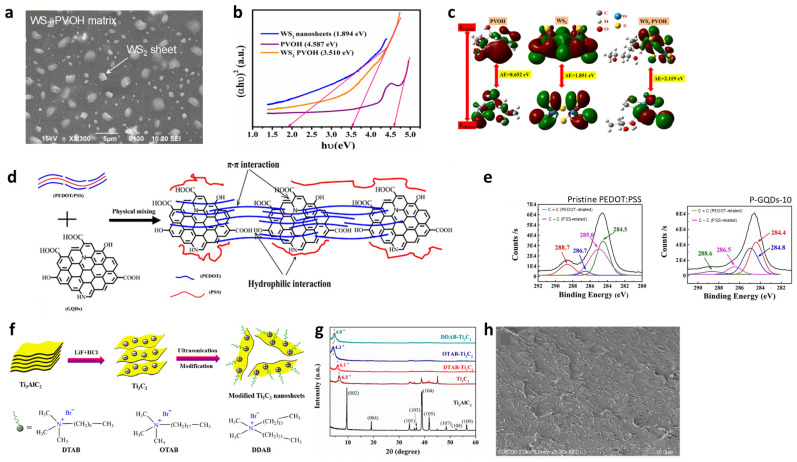
Two-dimensional–polymer matrix nanocomposites and interfacial interactions in embedded hybrid structures. (**a**) SEM image of WS_2_–PVOH nanocomposites showing uniformly dispersed WS_2_ nanosheets embedded within the polymer matrix. (**b**) Tauc plot demonstrating bandgap modulation upon hybridization: pristine WS_2_ (1.894 eV), PVOH (4.587 eV), and WS_2_–PVOH composite (3.510 eV). (**c**) DFT-calculated HOMO–LUMO charge distribution of WS_2_, PVOH, and WS_2_–PVOH hybrid, revealing interfacial charge transfer and the formation of new electronic states. Reproduced with permission from Ref. [24] (Royal Society of Chemistry, 2023). (**d**) Schematic illustration of dual-mechanism interfacial interaction between GQDs and the PEDOT:PSS matrix, highlighting the π–π conjugation with PEDOT and electrostatic interaction with PSS chains that promotes phase separation. (**e**) Deconvoluted C 1s XPS spectra of PEDOT:PSS and PEDOT:PSS–GQDs composites. Reproduced with permission from Ref. [43] (Springer Nature, 2018). (**f**) Synthesis and surfactant-assisted surface modification of Ti_3_AlC_2_ MXene nanosheets using cationic surfactants (DTAB, OTAB, DDAB) to improve compatibility with non-polar PS matrices. (**g**) XRD patterns showing (002) peak shifts in modified MXene samples, confirming interlayer expansion due to surfactant intercalation. (**h**) SEM image of OTAB-modified MXene–PS composite fracture surface. Reproduced with permission from Ref. [44] (MDPI, 2019).

Figure 2a is a SEM image of a WS_2_–PVOH composite, exhibiting uniformly distributed WS_2_ nanosheets into the PVOH matrix [24]. While the high crystallinity of WS_2_ is maintained, XRD and Raman analyses suggest that the interaction between the two materials is a weak electronic interaction predominantly at the hydrogen bonding level, rather than a strong covalent bond. Similar weak interactions mediated by hydrogen bonding have usually been reported to contribute to electronic or optical modulation in polymer hybrid systems, including GO and PVA composites [42]. Optical absorption measurements using a Tauc plot (Figure 2b) reveal a pronounced shift in the apparent optical transition energy of the WS_2_–PVOH hybrid compared to pristine WS_2_. To rationalize this modulation, the authors performed DFT simulations, which suggest that interfacial charge redistribution from WS_2_ to PVOH may occur, leading to modified electronic states at the interface (Figure 2c). While the DFT results support charge transfer as a contributing factor to the observed optical modulation, it should be noted that Tauc-derived optical shifts in 2D materials may also reflect contributions from other effects, such as excitonic transitions and environmental screening. Accordingly, careful interpretation is required when assigning the mechanisms underlying the observed bandgap change and charge transfer behavior.

Conversely, graphene quantum dot (GQDs)–PEDOT:PSS composite utilizes a strong, multi-component interfacial design that simultaneously forms strong π–π interactions with conductive PEDOT chains and engages in electrostatic interactions with the insulating PSS chains via functional groups of GQD and hydrophilic groups of PSS (Figure 2d) [43]. This robust and multiphase interaction induces phase separation of the PEDOT and PSS components and promotes the ordered alignment of the PEDOT chains on the GQD surface. Accordingly, the PEDOT surface enrichment resulting from PEDOT–PSS phase separation is corroborated by the XPS C 1s spectra (Figure 2e). For pristine PEDOT:PSS, the aromatic C=C com-ponent associated with PEDOT is located at 284.5 eV, while the aliphatic C–C component originating from the PSS chains appears at approximately 285.0 eV. Upon incorporation of GQDs, these carbon-related components exhibit discernible shifts, with the dominant C=C- and C–C-related contributions appearing at 288.6 eV and 286.5 eV, respectively. These pronounced core-level shifts are consistent with strong π–π interactions between PEDOT chains and GQDs, accompanied by interfacial charge redistribution at the PEDOT:PSS interface, rather than merely reflecting unchanged bonding configurations.

Beyond systems relying on inherent functional groups or π bonding, strong interfacial compatibility can also be engineered in otherwise incompatible systems through surface functionalization. In MXene (Ti_3_AlC_2_) and non-polar PS composites, strong adhesion is achieved by using cationic surfactants (DTAB, OTAB, DDAB), which electrostatically attach to the negatively charged Ti_3_C_2_ nanosheets and create a hydrophobic shell that acts as a coupling bridge for the non-polar PS matrix (Figure 2f) [44]. The success is structurally validated by the XRD patterns (Figure 2g), where a lower-angle shift of the (002) peak confirms surfactant intercalation and interlayer spacing expansion—direct proof of strong physical and electrostatic interaction. The enhanced interfacial adhesion is then visually confirmed by the fracture surface morphology, showing the OTAB-modified MXene nanosheets uniformly embedded within the PS matrix without aggregation (Figure 2h). Crucially, such uniform dispersion effectively mitigates the inherent oxidative instability of MXenes, as the polymer matrix provides barrier effects that effectively hinder the permeation of oxygen and heat [44]. This protective mechanism is quantitatively substantiated by a 25 °C increase in the initial decomposition temperature (*T*_5%_) and a 26.4% reduction in the peak heat release rate (PHRR) compared to the pure polymer, confirming that such hybridization substantially enhances the environmental and thermal stability of the resulting system.

### 2.2. Stacked or Layered Heterostructures

Stacked or layered 2D–polymer heterostructures integrate the polymer layer directly onto 2D materials, enabling device architectures in which the interfacial electrical coupling, rather than mechanical reinforcement, governs overall performance. Such vertically integrated polymer overlayers were initially introduced as gate dielectric materials, where their molecular flexibility, mechanical compliance, and low thermal budget processability provide distinct advantages over conventional inorganic oxides [45,46,47,48,49,50]. Moreover, the integration of 2D channels with ferroelectric polymers, such as P(VDF–TrFE) and P(VDF–TrFE-CFE), enables negative-capacitance FETs (NCFETs) with sub-60 mV/dec subthreshold swing [27,51,52,53]. However, the trend toward polymer-based NCFETs has largely diminished due to challenges in device stability, domain uniformity, and CMOS integration, redirecting ferroelectric polymers toward non-volatile memory applications rather than steep-slope switching, which will be discussed in the applications section. It is also worth noting that the interpretation of negative-capacitance continues to be discussed, particularly with respect to its polarization dynamics [54]. In parallel, ion-gel-based electric-double-layer (EDL) gating has also been widely explored, where the ultrahigh interfacial capacitance formed at the 2D–polymer interface enables sub-1 V operation and provides an additional low-voltage control route for 2D channels [55].

**Figure 3:**
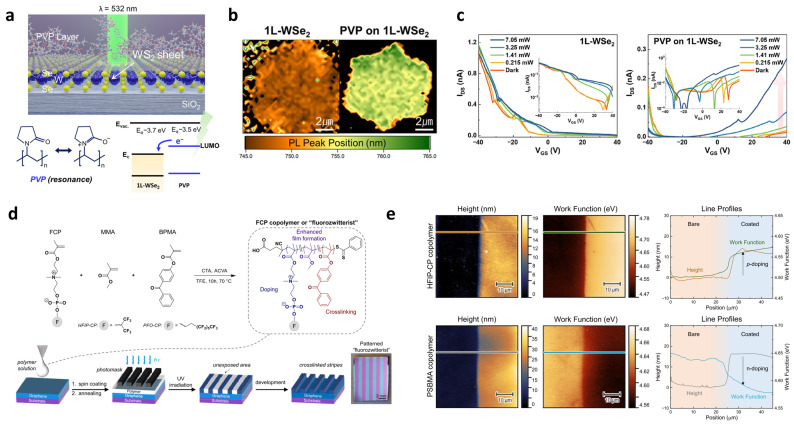
Stacked and layered 2D–polymer heterostructures. (**a**) Illustration of the PVP–1L–WSe_2_-stacked hybrid. The insets show the resonant structure of the PVP and electron transfer mechanism under light illumination. (**b**) PL peak position map for PVP-stacked WSe_2_ compared with WSe_2_. (**c**) Linear transfer curves for the 1L–WSe_2_ and PVP hybrid phototransistor under light injection, showing the dramatic p-to-n switching before and after PVP stacking. Reproduced with permission from Ref. [59] (American Chemical Society, 2024). (**d**) Synthetic scheme and photopatterning process of fluorinated zwitterionic copolymers (“fluorozwitterists”) for dipole-engineered work-function tuning. UV exposure induces crosslinking in selected regions, enabling spatial patterning of polymer dipoles directly on graphene substrates. (**e**) Atomic force microscopy (AFM) height images, Kelvin probe force microscopy (KPFM) work-function maps, and corresponding line profiles demonstrating localized electronic modulation induced by fluorozwitterist (HFIP-CP) and zwitterist (PSBMA) coatings. HFIP-CP generates p-doping (work-function increase), whereas PSBMA produces n-doping (work-function decrease), enabling the formation of laterally patterned p–i–n junctions within a single 2D sheet. Reproduced with permission from Ref. [62] (American Chemical Society, 2024).

As research has progressed, 2D–polymer-stacked systems have expanded beyond dielectric gating and evolved into active interfacial layers capable of directly modulating the electronic properties of 2D semiconductors [56,57,58]. For instance, Bang et al. reported a hybrid stacked structure consisting of bottom single-layer WSe_2_ (1L-WSe_2_) and top PVP, where the PVP layer acts as an electron source and trapping medium (Figure 3a) [59]. Here, the electron transfer is driven by the inherent difference in electron affinity and the presence of polar groups (C=O bonds) in the PVP structure, which facilitates the spontaneous donation of electrons to the WSe_2_ channel. This interfacial charge transfer results in pronounced n-type doping, as corroborated by several signatures, including a red-shift in PL (Figure 3b) and a transition of the field-effect characteristics from intrinsic p-type to clear n-type conduction (Figure 3c). Under illumination, PVP further serves as an electron-trapping overlayer, stabilizing photo-excited carriers and enabling persistent Fermi-level modulation for optoelectronic operation. Similarly, PEI has been widely utilized as a strong n-doping polymer for various 2D semiconductors, where its electron-rich amine groups efficiently donate electrons to the channel, yielding stable and robust n-type doping [57,58,60,61].

Beyond charge-transfer doping, recent studies demonstrate that stacked polymer layers can also serve as dipole-engineering platforms, enabling spatially programmable work-function and the energy-level modulation of 2D materials [62,63]. Pagaduan et al. engineered “fluorozwitterists”—fluorinated zwitterionic polymers with large built-in dipoles designed as negative-tone photoresists [62]. Through a lithographic sequence (coating → UV exposure → development), dipole-rich domains can be spatially patterned. The oriented dipoles induce bidirectional work-function modulation in the underlying graphene, enabling localized p- or n-type regions depending on dipole orientation (Figure 3d). Notably, co-patterning fluorozwitterist (HFIP-CP) and zwitterist (PSBMA) regions yields lateral p–i–n junctions within a single 2D sheet (Figure 3e), demonstrating that dipole alignment provides a powerful route to spatially programmable electronic-level architectures, beyond simple threshold voltage shifting.

### 2.3. Polymer-Functionalized 2D Hybrids

Polymer-functionalized 2D hybrids often employ strong chemical bonding, such as covalent grafting, to directly engineer the surface chemistry of 2D materials [64,65,66,67,68]. Unlike physical and weak intermolecular interactions, covalent modification creates permanent, electronically coupled interfaces that allow for precise control over electrical band alignment, environmental stability, and surface processability. However, covalent modification is challenging because the basal planes of many 2D materials are chemically inert with a lack of dangling bonds. Aggressive reaction conditions can generate structural disorder, reducing mobility, and increase undesired defect densities on the inert 2D surfaces. Consequently, effective functionalization strategies rely on exploiting intrinsic surface terminations, defects, or activated intermediates capable of reacting with the chemically stable 2D framework.

**Figure 4:**
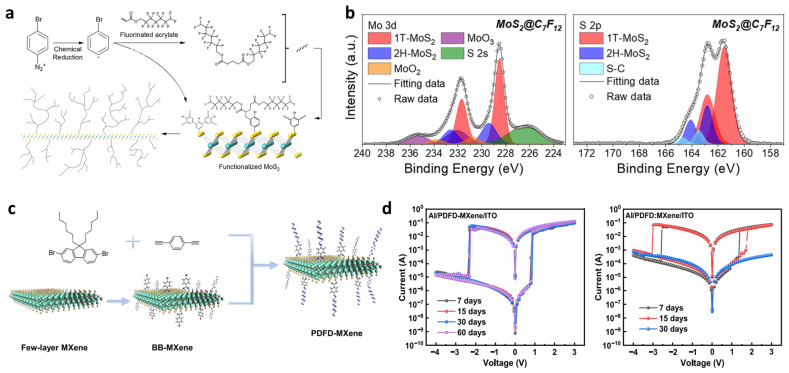
Surface functionalization of 2D materials and polymer-grafted structures. (**a**) Schematic illustration of surface functionalization of MoS_2_ nanosheets using chemically reduced diazonium salt followed by grafting of fluorinated acrylate chains. The reaction yields densely polymer-grafted MoS_2_, improving dispersion, interfacial compatibility, and chemical stability in polymer matrices. (**b**) XPS spectra of Mo 3d and S 2p core levels for functionalized MoS_2_@C_7_F_12_, showing contributions from 1T- and 2H-MoS_2_ phases, surface oxide species (MoO_2_, MoO_3_), and newly formed S–C bonding, confirming successful covalent grafting of fluorinated polymer chains. Reproduced with permission from Ref. [69] (American Chemical Society, 2021). (**c**) Synthesis route of polymer-grafted MXene (PDFD–MXene). Few-layer MXene is first reacted with a benzylic bromide linker (BB–MXene), enabling subsequent polymerization of the fluorinated monomer to yield a densely grafted and sterically stabilized MXene hybrid. (**d**) Electrical stability of Al/PDFD–MXene/ITO memristor devices measured over time, showing retention of bipolar resistive switching behavior for up to 60 days. The fluorinated polymer coating effectively suppresses oxidation and surface degradation, enabling long-term device reliability. Reproduced with permission from Ref. [70] (American Chemical Society, 2023).

Covalent polymer functionalization on TMDCs was achieved by Gómez-Muñoz et al., who developed a rapid and mild approach for coating MoS_2_ using diazonium-derived aryl radicals followed by in situ polymerization [69]. In this process, electron transfer from metallic 1T-MoS_2_ reduces aryl diazonium species to aryl radicals, which covalently graft onto the basal plane to form a phenylene anchoring layer. The same radicals simultaneously initiate the polymerization of fluorinated acryl monomers (Figure 4a), producing a dense polymer shell that is covalently bridged to MoS_2_ through S–C linkages, as confirmed by XPS (emergence of 163.5 eV S–C doublet) (Figure 4b). This approach achieves much higher surface coverage than conventional molecular grafting and yields tunable functional coatings, such as hydrophobic fluoropolymer layers with contact angles up to 150°, significantly improving the air-stability of MoS_2_. This strategy exemplifies the utility of radical-mediated grafting-from polymerization as a method for generating hybrid materials that provide both robust interfacial coupling and tunable surface functionality.

A grafting approach on other 2D materials was developed by Sun et al., who functionalized Ti_3_C_2_T_x_ MXene nanosheets using a conjugated polymer grafting-from reaction to fabricate high-performance non-volatile memory devices (Figure 4c) [70]. Here, 4-bromobenzoyl (BB) groups were first introduced onto few-layer MXene surfaces via nucleophilic substitution at surface –OH/–O terminations, forming aryl anchoring sites. These aryl groups served as reactive handles for surface-directed Sonogashira–Hagihara polymerization, enabling the growth of poly[(9,9-dihexyl-9H-fluorene)-alt-(1,4-diethynylbenzene)] (PDFD) chains directly from the BB–MXene surface. The resulting polymer-functionalized MXene displays dramatically improved solubility in organic solvents, enhanced oxidation resistance, and conformal polymer coverage. Importantly, the strong interfacial bonding facilitates controlled charge-transfer interactions between the conjugated polymer and MXene, yielding resistive switching with low turn-on voltage (0.46 V) and an ON:OFF ratio exceeding 10^4^. The covalent polymer layer also prevents MXene oxidation, enabling long-term device stability under humid conditions for approximately 60 days (Figure 4d), demonstrating how chemical anchoring can simultaneously enhance environmental robustness and introduce new charge-storage functionalities. Beyond the aryl-anchoring method, similar covalent grafting concepts have also been demonstrated in MXene systems. Their abundant surface terminations (–O, –OH, –F) enable in situ polymerization and surface-initiated polymer growth, including pyrrole [71,72] or ε-caprolactone [73] polymerization, as well as peroxide-initiated grafting [74] reactions. These studies collectively demonstrate that MXenes serve as a highly versatile platform for covalent polymer attachment, enabling various promising applications in sensor, catalyst, and energy technologies [75,76].

### 2.4. Polymer-Encapsulated 2D Layer

Polymer encapsulation offers a scalable and versatile strategy for enhancing the environmental and electrical stability of 2D materials by physically isolating the atomically thin channel from air, moisture, and charged adsorbates. A wide range of polymers, including PMMA [77,78,79,80,81], PVP [82], PVA [83], SU-8 [31], CYTOP [84,85], and parylene-C [86], have been explored to suppress hysteresis, reduce threshold voltage (*V*_TH_) shift, and improve long-term operational stability in 2D devices, especially FETs. However, their performance differs substantially depending on film permeability, crosslinking density, interfacial trap formation, and processing-induced damage.

**Figure 5:**
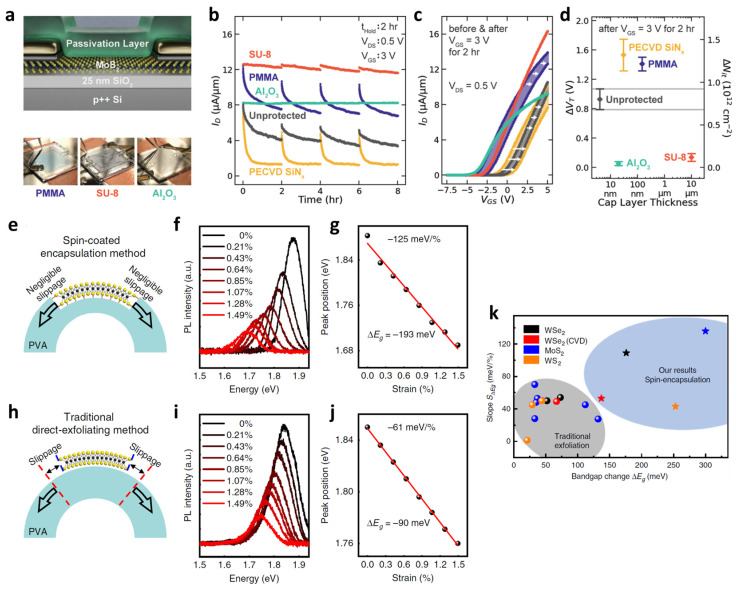
Polymer encapsulation on 2D materials. (**a**) Schematic illustration and optical images of MoS_2_ FETs encapsulated with PMMA, SU-8, and ALD-grown Al_2_O_3_. (**b**) Time-dependent drain current change under a 2 h positive gate-bias stress (*V_GS_* = 3 V, *V_DS_* = 0.5 V), showing that PMMA provides limited stabilization, whereas SU-8 and Al_2_O_3_ significantly suppress current degradation. (**c**) Transfer characteristics measured before and after the 2 h bias-stress test, highlighting the minimal threshold-voltage shift in SU-8 and Al_2_O_3_-capped devices compared to PMMA and unprotected channels. The white arrows indicate the direction of the threshold voltage shift. (**d**) Summary of threshold-voltage shift as a function of capping-layer thickness, demonstrating that SU-8 achieves stress-stability performance comparable to Al_2_O_3_. Reproduced with permission from Ref. [87] (American Chemical Society, 2020). (**e**) Schematic of strain transfer in PVA-encapsulated MoS_2_, enabling negligible interfacial slippage. The black arrows denote the direction of applied strain enabling negligible interfacial slippage. (**f**) PL spectra and (**g**) corresponding bandgap modulation under uniaxial strain for PVA-encapsulated MoS_2_. (**h**) Traditional direct-exfoliation transfer suffers from interfacial slippage. (**i**) PL shift and (**j**) corresponding bandgap modulation of non-encapsulated MoS_2_. (**k**) Comparison of bandgap modulation amplitude and modulation slope of polymer spin-encapsulation method with other traditional exfoliation of 2D materials on the flexible substrates. The five-pointed stars represent the results of the spin-encapsulation method from this work. Reproduced with permission from Ref. [90] (Springer Nature, 2020).

Doherty et al. conducted a systematic comparative assessment of various capping layers, including PMMA, SU-8, PECVD SiN_x_, and ALD-grown Al_2_O_3_, on MoS_2_ FETs (Figure 5a) [87].

Under 2 h positive gate-bias stress, PMMA-capped devices exhibited a time-dependent decay of drain current (*I*_D_) very similar to the behavior of unprotected devices (Figure 5b). The author mentioned that this insufficient stabilization is attributed to the relatively high permeability of PMMA and the formation of nanoscale air pockets at the 2D interface, which allow for the continued adsorption of environmental molecules. In contrast, the crosslinked polymer SU-8 exhibits markedly enhanced stress stability. Its densely crosslinked network forms a robust, low-permeability barrier that effectively blocks water and oxygen diffusion. SU-8-encapsulated devices maintain a higher drain current and display a minimal net threshold voltage shift (∆*V*_T_) compared to PMMA before and after the 2 h bias-stress test, demonstrating the advantage of rigid polymer networks over linear polymer films (Figure 5c). Figure 5d shows that SU-8 achieves the overall stress-stability window (∆*V*_T_) comparable to that of ALD-grown Al_2_O_3_, despite being processed entirely through a low-temperature spin-coating route. Therefore, SU-8 maintains this level of passivation while offering higher device yield and process simplicity than Al_2_O_3_, underscoring its practical advantage for scalable 2D integration.

Beyond stability, polymer encapsulation has also been recognized as a powerful strategy for enhancing the mechanical coupling between 2D materials and flexible substrates, which is crucial for efficient strain engineering [47,88]. The conventional transfer of 2D materials onto flexible substrates often leads to interfacial slippage, which severely limits strain transfer to the 2D lattice and reduces the efficiency of electronic and optical modulation [89]. A study by Li et al. demonstrated a breakthrough in strain engineering by using a simple spin-coating approach to encapsulate monolayer MoS_2_ within a flexible PVA film (Figure 5e) [90]. The strong interfacial interaction between the PVA and MoS_2_, combined with the high Young’s modulus of PVA (~10 GPa), ensures that the mechanical strain is effectively and uniformly transferred to the MoS_2_ lattice with negligible slippage. This mechanical coupling breakthrough resulted in a record-high bandgap modulation (∆*E*_g_) of −193 mV and a modulation rate of −125 meV/% under uniaxial strain (Figure 5f,g), which are significantly enhanced compared to the result of non-encapsulated MoS_2_ on the PVA substrate (Figure 5h–j). Figure 5k exhibits similarly large bandgap modulations in exfoliated and CVD-grown WSe_2_, demonstrating that spin-encapsulation using a polymer is a broadly applicable strategy for high-performance, strain-programmable 2D materials.

### 2.5. Fiber or Network Hybrid

Fiber- or network-based 2D–polymer hybrids utilize polymeric scaffolds, such as electrospun nanofibers [91,92,93], textile fibers [94], or porous polymer meshes [95], to integrate 2D materials into mechanically robust and deformable architectures. Unlike the previous configurations where 2D sheets are embedded or encapsulated within planar polymer matrices, fiber–network hybrids exploit three-dimensional pathways to form percolating conductive networks. These structures exhibit outstanding mechanical deformability and maintain electrical continuity even under bending, stretching, twisting, or repeated cyclic loading, making them highly attractive for wearable sensors [96,97,98] and energy storage devices [99,100] where conformability, breathability, and long-term mechanical durability are essential.

**Figure 6:**
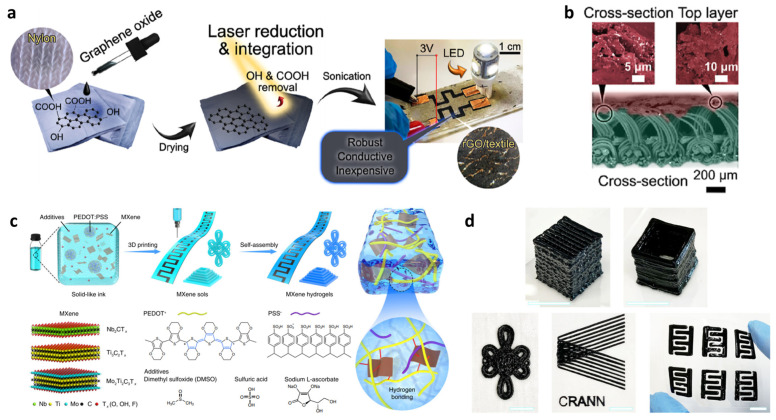
Fiber- and network-type 2D–polymer hybrid structures. (**a**) Schematic illustration of the fabrication of LIG–nylon textile hybrids. Graphene oxide is coated onto nylon fibers, dried, and subsequently reduced and integrated into the textile via laser irradiation, during which oxygen-containing groups are removed and rGO domains become embedded within the fiber surface. (**b**) Cross-sectional and top SEM images of the LIG–textile hybrid. Reproduced with permission from Ref. [101] (American Chemical Society, 2023). (**c**) Formation mechanism of MXene–PEDOT:PSS 4D-printed hydrogels. MXene–polymer inks undergo 3D printing and subsequent self-assembly, where PEDOT^+^–PSS^−^ interactions and additive-assisted gelation produce interconnected MXene networks within a hydrated polymer matrix. (**d**) Optical images of various 4D-printed MXene hydrogel architectures, including microlattices, hollow prisms, Chinese knots, center logos, and micro-supercapacitor arrays, demonstrating the geometric programmability and structural robustness of network-type 2D–polymer hybrids. The scale bars represent 1 cm. Reproduced with permission from Ref. [29] (Springer Nature, 2022).

Recent progress by Lipovka et al. demonstrates laser-induced graphene (LIG) and nylon textile composite for wearable textile sensors [101]. Here, GO is first coated onto woven nylon and subsequently reduced by laser irradiation, which simultaneously induces partial polymer softening and facilitates the interpenetration of reduced GO (rGO) domains into the textile fibers (Figure 6a). This laser-driven integration mechanism produces a multilayered conductive network conformal to textile geometry. Cross-sectional imaging reveals that rGO flakes embed into and anchor along the fiber bundles, resulting in well-intermixed conductive textiles (Figure 6b). Because the conductive pathways follow the natural anisotropy of the textile weave, the hybrid maintains electrical continuity under bending, stretching, or vibration, enabling applications in muscle-motion detection, voice recognition, and pulse monitoring.

In addition, Li et al. developed 4D-printed features using MXene–PEDOT:PSS hydrogels that self-assemble into porous, interconnected MXene networks [29]. Their ink formulation enables the 3D printing of MXene sols, followed by a mild thermal activation step that drives PEDOT^+^ chains to interact strongly with MXene surfaces through hydrogen bonding, electrostatic attraction, and π–π coupling (Figure 6c). The concurrent removal of insulating PSS^−^ chains further strengthens MXene–polymer contacts. The resulting hydrogels possess a continuous three-dimensional conductive framework, with MXene sheets functioning as high-conductivity channels and PEDOT:PSS acting as a flexible, chemically coupled binder. This architecture supports the fabrication of diverse geometries, including microlattices, hollow prisms, Chinese knots, center logos, and micro-supercapacitor arrays, while retaining excellent mechanical integrity and high-quality electrochemical performance.

**Table 1:** Representative 2D–polymer hybrid architectures classified by hybrid configuration (embedded, stacked, functionalized, encapsulated, and networked), highlighting their dominant interfacial interaction mechanisms and the resulting impacts on charge transport, mechanical stability, device functionality, and performance.

Hybrid Structure	Materials	Hybrid Method	Dominant Interfacial Interaction	Charge Transport Impact	Mechanical Stability	Applications	Device Performance	Ref.
Polymer-embedded 2D sheets	GO–PEO/LiTFSI	Solution casting of GO–PEO–LiTFSI mixture	Hydrogen	GO suppresses PEO crystallization, increasing amorphous regions for Li^+^ transport	Tensile stress: 1.31 MPa (1356% increase), Toughness: 1768.4 kJ/m^3^	All-Solid-State Lithium Metal Battery	Ionic conductivity: 1.54 × 10^−5^ S/cm	[28]
	RGO–Co_3_O_4_–P(VDF)	In situ growth, Hot-press	Interfacial dipole	Dielectric loss increases with filler loading	Flexible as pure P(VDF)	Microwave absorption, EMI shielding	RL-25.05 dB at 11.6 GHz	[37]
	Graphene–SIS	Solution mixing, Evaporation-induced self-assembly	π–π interaction	Surface resistivity decreased by four orders of magnitude	Tensile strength + 26.4% (at 0.5 wt%), Hardness + 25.7%	Photomechanical actuation, Thermal management	Thermal conductivity increased by 42%	[39]
	MoSe_2_–PS-NH_2_	Solvent evaporation	Lewis acid–base	Polymer-assisted vertical percolation of conducting MoSe_2_	Stable flexibility (1000 bending cycles)	Flexible photodetectors	Responsivity up to 2.5 A W^−1^, Detectivity ~10^14^ Jones,	[102]
Stacked or layered heterostructure	BP–MoS_2_–P(VDF–TrFE)	Spin-casting and thermal annealing of ferroelectric polymer on 2D flakes	Interfacial dipole	C–F dipoles induce hole accumulation, suppress electron injection	Polymer coating retards degradation in ambient air (passivation effect)	Non-volatile Ferroelectric Memory (FeFET), CMOS Inverter	Memory window: ~15 V, Mobility: 1159 cm^2^/Vs, ON–OFF: 10^5^	[26]
	WSe_2_ –CYTOP	Spin coating of fluoropolymer buffer layer followed by top-gate fabrication	Electrostatic interaction	Interface trap reduction, contact barrier modulation	Stable operation in air over weeks	p-channel WSe_2_ FETs	Hole mobility up to ~200–250 cm^2^ V^−1^ s^−1^, ON–OFF ratio ~10^6^, subthreshold swing ~60–100 mV dec^−1^	[85]
	SF@MXene	Composite membrane	Hydrogen bonding	metallic conductivity on fibrous structures, ensuring stable charge transport	Elastic modulus 1.22 MPa	Flexible pressure sensor	Sensitivity 25.5 kPa^−1^ response–recovery time 40/35 ms	[96]
	MXene–ePPO	interfacial bonding	Hydrogen bonding interaction	pathways for Li+ by Lewis acid-based interaction	Young’s modulus 10.5 Mpa	Battery	Ionic conductivity 4.6 × 10^−4^ S cm^−1^	[103]
Polymer-functionalized 2D hybrid	MoS_2_–Poly(TPARA-co-EDOT)–Peptide	Peptide-imprinted electropolymerization on MoS_2_ monolayer	van der Waals	enhanced electrochemical current response	Reusability confirmed (six cycles)	Biosensor (MMP-1 lung cancer biomarker)	LOD: 1.0 fg/mL, Accuracy: 95% vs. ELISA	[23]
	BP–Aryl diazonium	Covalent functionalization	Covalent	Controllable p-type doping, enhanced hole mobility	Air stability > 3 weeks	Field-effect transistors (FETs)	ON–OFF ratio 10^6^, Mobility ~150 cm^2^ V^−1^ s^−1^	[30]
Polymer-encapsulated 2D layer	MoS_2_–PANI	In situ polymerization of hydrothermally synthesized MoS_2_ with PANI	van der waals	PANI conductive pathways, band alignment facilitates electron–hole separation	Flexible substrates mentioned	Broadband Photodetector	Photoresponsivity: 25 A/W (@785 nm), QE: 38.21%	[25]
	MoS_2_–PI	vdW transfer of MoS_2_ onto solution-processed polyimide substrate	van der Waals	Polymer-assisted preservation of continuous MoS_2_ conduction pathways	Stable up to 1000 bending cycles	Flexible electronics, wearable FETs	ON–OFF ratio ~10^7^, field-effect mobility ~30–40 cm^2^ V^−1^ s^−1^	[47]
	MoS_2_–CYTOP	Spin coating of CYTOP passivation layer	Electrostatic interaction	Suppression of charge trapping, hysteresis in MoS_2_ channel	Stable electrical characteristics after >10^4^ s bias stress	MoS_2_ field-effect transistors	Hysteresis reduced from ~10–15 V → ~2–3 V	[84]
	MoS_2_–(PMMA, CYTOP)	Spin coating of polymer capping layers on MoS_2_ FETs	Electrostatic interaction	Suppression of charge trapping, bias-stress-induced carrier instability	Electrical stability maintained during >10^4^ s gate bias stress	MoS_2_ field-effect transistors	Threshold-voltage shift reduced by ~50–80%	[87]
	MoS_2_–polyvinyl formal	Spin coating of Formvar encapsulation layers	Electrostatic interaction	Strain-induced bandgap, carrier transport modulation	Stable strain transfer over >100 strain cycles	Strain-engineered optoelectronics	Strain transfer efficiency enhanced by ~2×; photoluminescence modulation >50%	[88]
	(MoS_2_, WS_2_, WSe_2_, graphene)–(SU-8, PMMA)	Spin coating of polymer encapsulation layers	Electrostatic interaction	Strain-induced band structure, carrier transport modulation	Reversible strain transfer maintained up to ~2% strain over multiple cycles	Strain-engineered electronic and optoelectronic devices	Strain transfer efficiency > 90%	[90]
Fiber or network hybrid	MXenes–PEDOT:PSS	4D printing (Heat-stimulated self-assembly)	Electrostatic, Hydrogen	High electrical conductivity, fast ion transport	Robust integrity after vigorous shaking	Supercapacitors, Micro-supercapacitors	232.9 F g^−1^ capacitance, 92.88 μWh cm^−2^ energy density	[29]
	PVA-GO	Solution-casting	Hydrogen	Conductive network formation	Tensile strength + 225%, Elongation + 37.16% (at 0.5 wt%)	Strain sensing, Energy storage	Gauge factor 2.46, Specific capacitance 124.7 F/g	[42]
	MXene–Polyester textile fibers	Dip-coating-based MXene deposition on textile fibers	Electrostatic interaction	Conductive fiber network formation	Maintained EMI performance after 20 washing cycles and repeated bending	Joule heating textiles	Joule heating temperature ~100–150 °C at low voltage	[72]
	MXene–TPU	filtration assisted self-assembly	Hydrogen bonding	Conductive network allowing for sensitive resistance	Stability and recoverability (2600 cycles)	Strain sensor	Gauge factor 37.5 in 0−50%	[97]
	rGO–nylon	Laser reduction, integration	hydrogen bonding	Resistance variation upon mechanical stimulus	Elongation at break ~881%	Gesture sensor, electronalization	Sheet resistance 87.6 ± 36.2 Ω/sq	[101]
	PEDOT:PSS–rGO–MoS_2_	Co-assembly	π−π interaction	MoS_2_ pseudocapacitance, GO ion diffusion, PEDOT:PSS conductivity	Capacitance retention (1000 bending cycles)	Supercapacitor	Volumetric specific capacitance 325.8 F cm^−3^	[104]

## 3. Fabrication Strategies of 2D–Polymer Hybrid Structures

The formation of 2D–polymer hybrid structures relies heavily on processing routes that can preserve the intrinsic properties of 2D materials while establishing controlled interfaces with polymer. This section outlines the key processing approaches used to realize the hybrid architectures introduced in Section 2, highlighting how different fabrication routes govern dispersion quality, interfacial coupling, and structural fidelity across various device platforms.

### 3.1. Physical Dispersion and Solution Casting Methods

The most basic and widely used strategy to combine 2D materials with polymers is a solution-based physical dispersion method [28,105,106,107,108,109,110,111,112]. In this approach, the two components are homogeneously mixed in a liquid medium and then processed into composite films by casting [28,109,112,113]. The resulting composites are stabilized mainly by non-covalent interactions [28,105], such as hydrogen bonding; electrostatic interactions; and van der Waals forces. Although these interactions inherently limit the interfacial binding strength and electronic coupling across the interface, they offer advantages in terms of preserving the structural integrity of 2D materials through minimal material damage [112] and high process flexibility [28]. Owing to its conceptual simplicity, low cost, and broad compatibility with diverse 2D material–polymer systems, this solution dispersion route has become a fundamental yet highly practical platform for realizing 2D–polymer hybrid structures.

**Figure 7:**
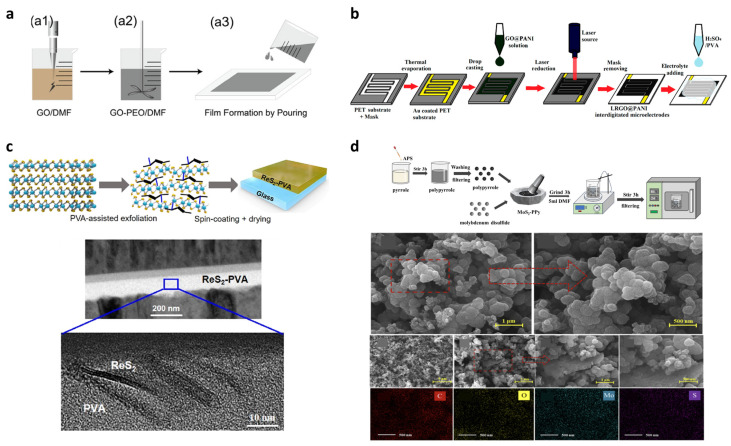
Physical dispersion and solution casting methods. (**a**) Schematic illustration of the fabrication of a solution-dispersed GO–PEO solid polymer electrolyte via simple casting and DSC curves showing suppressed PEO crystallinity upon GO incorporation. Reproduced with permission from Ref. [28] (American Chemical Society, 2021). (**b**) GO–PANI hybrid via dispersion laser-assisted reduction. Reproduced with permission from Ref. [105] (American Chemical Society, 2020). (**c**) ReS_2_-PVA nanocomposite via polymer-assisted exfoliation. Reproduced with permission from Ref. [106] (American Chemical Society, 2021). (**d**) PPy–MoS_2_ film via mechanical blending. Reproduced with permission from Ref. [113] (MDPI, 2024).

Figure 7a summarizes the fabrication process of a GO–PEO-based solid polymer electrolyte (SPE) reported by Wen et al. [28] The fabrication consists of three steps. In step (a1), GO is exfoliated and dispersed in DMF by ultrasonication. In step (a2), PEO and LiTFSI are added to the GO–DMF dispersion to form a homogeneous slurry. The –OH and –COOH functional groups of GO form hydrogen bonds with the ether oxygen of PEO, thereby tuning chain orientation and miscibility. Finally, in step (a3), the slurry is solution-cast onto a substrate and dried, yielding an SPE film in which GO is uniformly distributed. The right side of Figure 7a further presents differential scanning calorimetry (DSC) curves of pristine PEO, PEO–LiTFSI, and GO–PEO electrolytes, providing direct thermal evidence of suppressed PEO crystallization upon GO incorporation. The reduced melting (*T*_m_) and glass transition (*T*_g_) temperatures indicate an increased amorphous fraction in the GO–PEO matrix, which is favorable for Li^+^ transport. Together, these results demonstrate that non-covalent GO–PEO interactions contribute not only to structural stabilization of the composite film but also to enhanced ionic transport through crystallinity suppression.

Beyond simple casting methods, a laser-reduced GO@PANI (LRGO@PANI) strategy provides a more advanced form of solution-processed hybridization by integrating in situ polymer growth on GO with localized laser reduction [105]. Here, GO@PANI hybrid dispersion is drop-cast onto pre-patterned Au microelectrodes. Subsequent selective laser irradiation reduces GO to highly conductive LRGO, simultaneously creating fine patterns and defining conductive pathways within the PANI network. After mask removal and the deposition of a solid-state electrolyte (H_2_SO_4_–PVA), the resulting electrode integrates the conductive network of LRGO, the pseudocapacitance of PANI, and the ion-accessible porous structure for great micro-supercapacitor characteristics. Moving beyond direct mixing, polymer-assisted exfoliation offers another efficient strategy for hybrid formation, as demonstrated in Figure 7c [106]. Here, PVA chains intercalate into the interlayer gaps of ReS_2_, during sonication, weakening van der Waals interactions and promoting exfoliation. The exfoliated nanosheets remain embedded within the PVA matrix after spin-coating, forming a layered hybrid architecture confirmed by cross-sectional TEM image below Figure 7c. This structural configuration enables stable separation of ReS_2_ nanosheets and controlled trap-site participation, leading to an enhanced and persistent photoresponse. Importantly, the polymer matrix plays a stabilizing role by suppressing environmental degradation, thereby improving reproducibility and switching stability in memristive operation. Overall, this process represents a simple yet efficient strategy for implementing high-quality 2D–polymer hybrids in a solution, combining surfactant-free aqueous exfoliation with polymer-assisted stabilization.

Finally, Figure 7d highlights a solid-state blending route in which p-type polypyrrole (PPy) and n-type MoS_2_ powders are mechanically co-milled to form a binary hybrid [113]. The fabrication consists of two main steps. First, pyrrole is oxidatively polymerized using ammonium persulfate (APS) to produce PPy powder. Second, PPy powder and layered MoS_2_ powder are co-milled to form a composite in which MoS_2_ nanosheets intercalate between the granular PPy particles. This composite powder is then dispersed in DMF to form a slurry, which is deposited by drop casting onto silver fork-finger electrodes on a PI substrate, yielding a porous 2D–polymer layer. SEM and EDS analyses confirm hybrid morphology and elemental uniformity. Although chemically simple, this approach yields complementary p-type–n-type conduction and porous transport channels that are particularly effective in gas sensing platforms.

### 3.2. Solution-Mediated In Situ and Chemical Polymerization

In solution-mediated approaches, 2D materials and polymers form hybrid structures through direct polymerization, adsorption, and ordering in a liquid environment [32,114,115]. Unlike simple physical mixing of preformed components, these methods rely on intrinsic chemical mechanisms in which polymer chains grow in situ on 2D surfaces or reactive species formed in solution selectively adsorb and align on the 2D substrate [32,114,116], while interfacial interactions and electronic structures are established concurrently. Such surface-directed polymerization and chemical functionalization exploit the defect-free van der Waals surfaces of 2D materials as reaction platforms, effectively preserving the structural integrity of the 2D materials while enabling precise control over polymer growth [115], backbone orientation [104], interfacial dipole formation [117], and charge-transfer coupling [32]. As a result, 2D–polymer heterostructures produced by these methods can exhibit high interfacial quality with fundamentally improved interfacial stability [117], continuous charge transport pathways [104], and optoelectronic functionality [32] compared to physically mixed composites.

**Figure 8:**
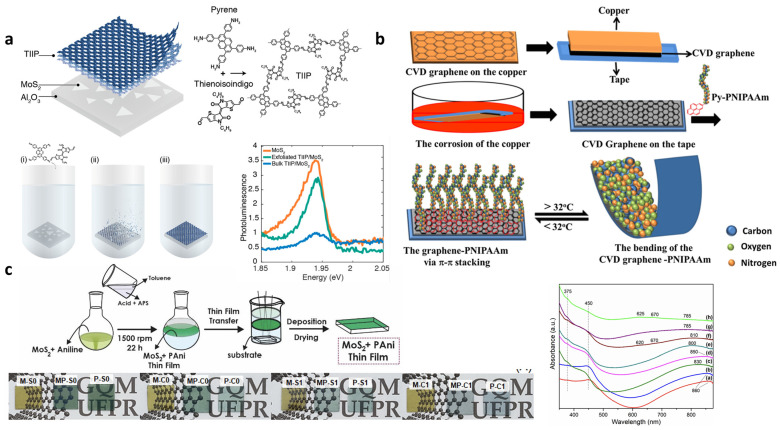
Solution-mediated in situ and chemical polymerization. (**a**) Solvothermal in situ polymer growth on 2D MoS_2_ film and PL spectra of pristine MoS_2_ and TIIP–MoS_2_ heterostructure with different TIIP thicknesses. Reproduced with permission from Ref. [32] (American Chemical Society, 2020). (**b**) Solution-phase non-covalent polymer functionalization of graphene film. Reproduced with permission from Ref. [114] (ELSEVIER, 2017). (**c**) Interfacial in situ polymerization of PANI on MoS_2_ nanosheet. In bottom figure, M-S0: MoS_2_ film in H_2_SO_4_ at pH 0, MP-S0: MoS_2_-PANI film in H_2_SO_4_ at pH 0, P-S0: PANI film in H_2_SO_4_ at pH 0, M-C0: MoS_2_ film in HCl at pH 0, MP-C0: MoS_2_-PANI film in HCl at pH 0, P-C0: PANI film in HCl at pH 0, M-S1: MoS_2_ film in H_2_SO_4_ at pH 1, MP-S1: MoS_2_-PANI film in H_2_SO_4_ at pH 1, P-S1: PANI film in H_2_SO_4_ at pH 1, M-C1: MoS_2_ film in HCl at pH 1, MP-C1: MoS_2_-PANI film in HCl at pH 1, P-C1: PANI film in HCl at pH 1. In UV–Visible: (a) P-S0, (b) MP-S0, (c) P-C0, (d) MP-C0, (e) P-S1, (f) MP-S1, (g) P-C1, (h) MP-C1. Reproduced with permission from Ref. [115] (American Chemical Society, 2025).

Figure 8a shows the formation of a 2D–polymer hybrid (TIIP-2DP) that was directly grown on monolayer MoS_2_ through surface-templated solvothermal polymerization [32]. The upper-right panel schematically illustrates the molecular structures of the monomers (pyrene-based diamine and thienoisoindigo-based dialdehyde), the bonding network of the resulting TIIP-2DP, and the direct solvothermal polymerization procedure on the monolayer TMDC surface. The ordered and dangling-bond-free CVD-grown MoS_2_ surface serves as an ordered template that guides monomer adsorption and promotes in-plane polymerization, resulting in a continuous π-conjugated 2DP sheet with well-defined interfacial geometry. The bottom-right side of Figure 8a presents the PL response of MoS_2_ after TIIP-2DP integration. Compared to pristine monolayer MoS_2_, the TIIP-2DP–MoS_2_ heterostructure exhibits a pronounced quenching in MoS_2_ PL intensity, indicating the emergence of an additional non-radiative decay pathway at the interface. This PL quenching is attributed to interfacial energy transfer from MoS_2_ to the π-conjugated TIIP-2DP layer, enabled by the intimate interfacial contact formed during surface-directed polymerization. Importantly, when considered together with the well-established picosecond-scale exciton lifetime of monolayer MoS_2_, the high quenching efficiency (reaching ~85%) implies an ultrafast interlayer energy transfer process, supporting the presence of efficient interfacial electronic interaction rather than a simple physical proximity effect.

A complementary solution-mediated functionalization mechanism is shown in Figure 8b, where CVD graphene is non-covalently modified with pyrene-terminated poly(N-isopropylacrylamide) (PNIPAAm) [114]. The pyrene units selectively adsorb onto the exposed graphene surface via π–π stacking, enabling one-side functionalization after graphene transfer from Cu foil to tape substrate. Because PNIPAAm exhibits a thermally induced volume change near its lower critical solution temperature (LCST), the asymmetric polymer layer induces bending of the hybrid film as the temperature varies. Above the LCST, PNIPAAm chains dehydrate and contract, leading to a shortening of the top layer and resulting in bending of the composite. Below the LCST, the chains rehydrate and swell, releasing the strain and restoring a flat configuration. This simple π-stacking-based functionalization therefore produces a thermo-responsive actuator without covalent chemistry or complex processing.

Figure 8c shows an interfacial in situ polymerization route for forming MoS_2_–PANI thin films at the liquid–liquid boundary [115]. First, MoS_2_ nanosheets are dispersed in acetonitrile, and aniline is added to form a precursor mixture. This mixture is then introduced into a biphasic system consisting of a strongly acidic aqueous phase and toluene. In the aqueous phase, aniline becomes protonated and thereby more reactive, while oxidants near the interface drive interfacially confined 2D polymerization of aniline. As a result, PANI chains grow parallel to the interface, naturally forming thin, continuous PANI 2D films.

During polymerization, MoS_2_ nanosheets are captured and aligned within the growing PANI network through π–π interactions and electrostatic interactions with charged species. The hybrid films transferred onto solid substrates show different colors depending on acid type and pH due to changes in the degree of protonation. Under strongly acidic conditions, the conversion from benzenoid to quinoid structures and the formation of polaron and bipolaron states are promoted, increasing the doping level. These effects manifest in the UV–Vis spectra as growth of the polaron band around ~450 nm and the bipolaron band near ~800 nm, accompanied by a deepening of the film color. These optical signatures confirm efficient electronic interactions between MoS_2_ and PANI, which contribute to enhanced charge transport and electrochemical activity in the resulting composite.

### 3.3. Vapor-Phase and Transfer-Mediated Interfacial Assembly

Approaches in which polymers are directly deposited onto 2D films either by interfacial processes [118,119,120] or vapor-phase polymerization [121,122,123] offer a level of precise control that distinguishes them from solution-dispersion-based methods. In these systems, monomer, oxidant, or initiator vapors react directly at the 2D surface, ensuring high interfacial quality by allowing for fine tuning of film continuity, thickness, composition, doping level, and interfacial uniformity without constraints imposed by solvent wetting or surface energy [122]. Moreover, interfacial dipoles [118], charge-transfer interactions [119], polymer-induced passivation effects [121], and optoelectronic coupling [118,119] generated during deposition can be intentionally engineered. Consequently, vapor-phase and interfacial deposition provide a powerful platform for designing high-performance 2D–polymer hybrids with actively tunable electrical and optical responses.

**Figure 9:**
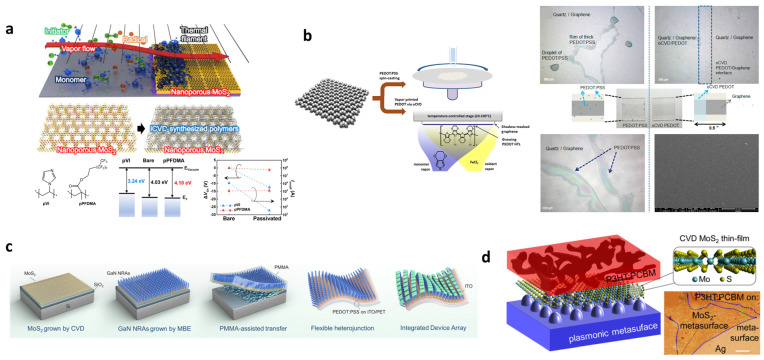
Vapor-phase and transfer-mediated interfacial assembly. (**a**) iCVD passivation of nanoporous MoS_2_ with conformal pVI and pPFDMA coatings enabling tunable doping and environmental stability. The bottom panels (from left to right) show the molecular structures of pVI and pPFDMA, the work-function modulation of MoS_2_ induced by polymer passivation, and the corresponding changes in threshold voltage and ON–OFF ratio before and after polymer passivation. Reproduced with permission from Ref. [121] (Springer Nature, 2022). (**b**) Comparison of spin-coated PEDOT:PSS and oCVD PEDOT films on graphene, highlighting uniform vapor-deposited polymer layers on hydrophobic 2D surfaces. Reproduced with permission from Ref. [122] (American Chemical Society, 2012). (**c**) Transfer mediated interfacial integration of CVD MoS–GaN nanorod heterostructures onto PEDOT:PSS-coated flexible substrates. Reproduced with permission from Ref. [118] (Springer Nature, 2024). (**d**) Formation of P3HT:PCBM–MoS_2_ heterojunctions on plasmonic metasurfaces using transferred monolayer MoS_2_ and solution-cast organic layers. Reproduced with permission from Ref. [119] (American Chemical Society, 2016).

Figure 9a shows the initiated CVD (iCVD)-based passivation of nanoporous MoS_2_ [121]. When monomer and initiator vapors are introduced to nanoporous MoS_2_, monomers first adsorb on the low-temperature substrate surface, and radicals generated from a heated filament initiate polymerization directly on the adsorbed monomer layer, forming a conformal polymer coating. Because iCVD relies on surface adsorption rather than line-of-sight flux, it can uniformly coat the inner surfaces of porous architectures. Two types of polymer passivation layers are employed. The first, poly(1-vinylimidazole) (pVI), is an electron-donating polymer based on an imidazole ring that induces strong n-type doping in MoS_2_ by donating electrons. UPS analysis shows a reduction in the work function from 4.03 eV to 3.24 eV. Correspondingly, pVI-passivated devices exhibit a significant negative threshold voltage shift (∆*V*_TH_) in their transfer characteristics. This response stems from an increased electron density in the MoS_2_ channel rather than intrinsic mobility changes, offering complementary evidence of the interfacial electronic-level modulation induced by pVI. The second, poly(1H,1H,2H,2H-perfluorodecyl methacrylate) (pPFDMA), is a highly hydrophobic polymer with a long fluoroalkyl chain. It has little effect on doping (work function ~4.10 eV) and accordingly induces negligible shifts in ∆*V*_TH_, indicating minimal perturbation of the channel carrier density. Instead, pPFDMA effectively suppresses O_2_ and H_2_O adsorption, significantly improving the electrical stability and environmental robustness of nanoporous MoS_2_.

Another approach using oxidative CVD (oCVD) was also reported by Park et al. [122] Figure 9b shows a comparison of spin-coated PEDOT:PSS films and oCVD-grown PEDOT films on graphene. Spin-coated PEDOT:PSS suffers from severe dewetting on the hydrophobic graphene surface, leading to the formation of droplets, rims, and discontinuous films. In contrast, oCVD generates uniform conformal PEDOT layers through direct vapor-phase oxidative polymerization of EDOT on graphene. This solvent-free vapor-phase process does not rely on solution wetting and therefore produces uniform coatings even on highly hydrophobic graphene. The deposition temperature (~120 °C) is low enough to preserve the structural integrity of the 2D layer by avoiding damage or deformation of the graphene. Moreover, the work function of oCVD PEDOT can be precisely tuned in the range of ~4.9–5.2 eV by controlling the Cl^−^ doping level, which helps compensate for the intrinsically low work function of graphene and improves energy-level alignment with donor polymers at the interface.

Beyond direct vapor polymerization, hybrid interfaces can also be formed through transfer-mediated processes that rely on pre-deposited polymer layers. Figure 9c shows the fabrication of MoS_2_ and GaN nanorod arrays, which are laminated onto a PEDOT:PSS-coated flexible substrate using PMMA-assisted transfer [118]. Although PEDOT:PSS is solution-cast, the resulting 2D–polymer interface governs charge transport and mechanical integration, demonstrating that polymer-coated substrates can act as active interfacial layers during 2D material transfer.

A related work is shown in Figure 9d, where CVD-grown monolayer MoS_2_ is transferred onto plasmonic metasurfaces before spin-coating the P3HT:PCBM active layer [119]. Because monolayer MoS_2_ exhibits a dangling-bond-free 2D van der Waals surface, it forms a largely trap-free, stable non-covalent interface with the P3HT:PCBM layer. Within the active layer, P3HT serves as the electron donor and PCBM as the electron acceptor, while the n-type MoS_2_ adds an additional electron-transport component. Together, these materials form a p–n-type van der Waals heterojunction at the 2D–polymer interface, enabling efficient charge separation and transfer. Enhanced light absorption by the plasmonic metasurface further increases photogeneration, thereby improving the overall optoelectronic performance of the device. This example highlights how interfacial polymer deposition can be combined with photonic engineering to boost device performance.

## 4. Emerging Device Applications

The diverse structural motifs and tunable interfacial interactions enabled by 2D–polymer hybrid architectures open pathways to a broad range of advanced device applications. By combining the chemical functionality, mechanical compliance, and processability of polymers with the electrical, optical, and surface characteristics of 2D materials, these hybrids offer synergistic properties that are difficult to achieve with either material classes alone. Their engineered interfaces formed through various methods mentioned in Section 3 allow for precise control over charge transport, molecular recognition, exciton coupling, and ionic–electronic interactions. Owing to these synergistic attributes, 2D–polymer hybrids are emerging as versatile platforms for chemical sensors, optoelectronic devices, neuromorphic memory systems, and energy devices.

### 4.1. Chemical Sensors

Two-dimensional–polymer hybrid heterostructures have emerged as highly effective sensing platforms owing to their synergistic combination of the intrinsic surface activity of 2D materials and the chemical tunability of polymers. Such hybrids offer enhanced sensitivity, selectivity, and mechanical compliance that are increasingly essential for next-generation chemical and biological sensing technologies [107,113,124]. Among these, gas sensors have been most extensively studied due to the strong surface reactivity of 2D materials and the selective response of polymers.

**Figure 10:**
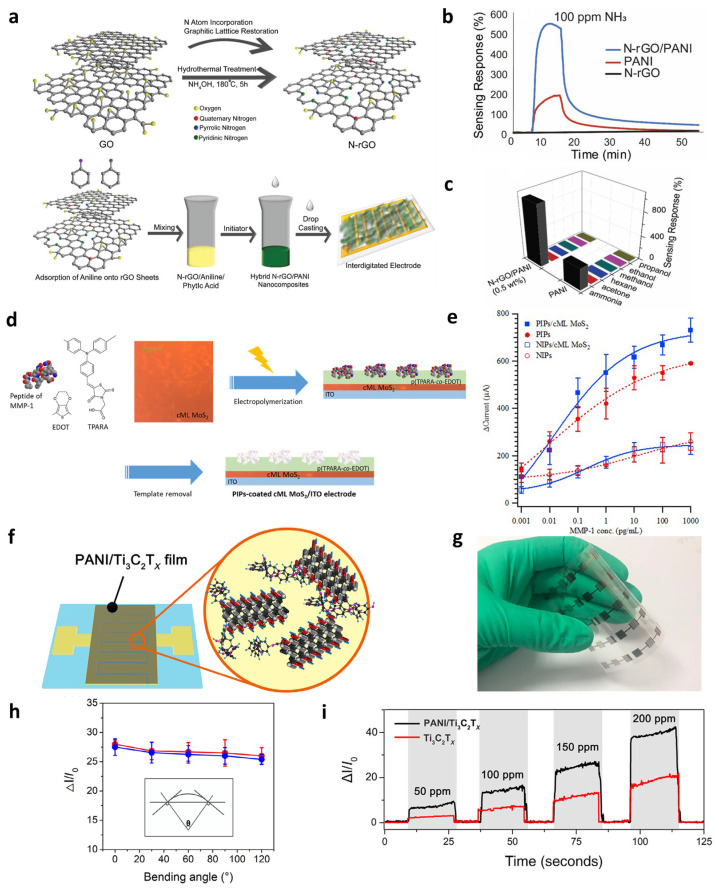
Performance and mechanisms of hybrid sensors based on 2D materials and polymers. (**a**) Schematic illustration of the fabrication process of N-rGO and N-rGO–PANI nanocomposite. (**b**) Comparison of the sensing response of pristine PANI, N-rGO nanosheet, N-rGO–PANI nanocomposite toward 100ppm of NH_3_. (**c**) Comparison of gas selectivity between N-rGO–PANI and pristine PANI sensors. Reproduced with permission from Ref. [109] (Wiley-VCH, 2019). (**d**) Schematic illustration of PIPs-coated cML MoS_2_/ITO electrode. (**e**) Sensing performance curves of four different sensor configurations. Reproduced with permission from Ref. [23] (Elsevier, 2023). (**f**) Schematic of the Au electrode coated with PANI–Ti_3_C_2_T_x_ nanocomposites. (**g**) Photograph of the fabricated device on a PET substrate and (**h**) the response change under different angles (0º~120º), where the red and blue curves represent the flat state and bent state. (**i**) Sensitivity measured as the concentration of the target molecule increases in pristine MXene and the nanocomposite. Reproduced with permission from Ref. [112] (Wiley-VCH, 2019).

Tanguy et al. [109] demonstrated this synergy using an N-doped rGO (N-rGO)–PANI nanocomposite for NH_3_ gas detection (Figure 10a–c). The fabrication process involves N doping of GO followed by formation of an N-rGO–PANI hybrid via oxidative polymerization (Figure 10a). The two components form effective molecular interactions, in which the high conductivity of N-rGO and the gas response of PANI act synergistically to facilitate charge transport and enhance the electrical signal upon gas adsorption (Figure 10b). Consequently, an enhancement of about 2.8-fold in sensing performance and 20% in recovery ratio was achieved relative to pristine PANI. Furthermore, the sensor demonstrates strong selectivity toward NH_3_ over common interfering gases, indicating that the polymer chemistry of PANI plays a dominant role in discriminating the target analyte, while the N-rGO network effectively transduces these selective interactions into amplified electrical signals (Figure 10c).

Beyond gas sensing, peptide-imprinted polymers integrated with 2D semiconductors have enabled highly selective biosensing [125]. Lee et al. [23] employed a peptide-imprinted poly(TPARA-co-EDOT) coating on continuous monolayer MoS_2_ to detect the matrix metalloproteinase-1 (MMP-1) peptide (PIPs/cML-MoS_2_) (Figure 10d). The molecularly imprinted film provides specific binding cavities for the MMP-1 peptide, while the cML-MoS_2_ layer ensures efficient transduction. As shown in Figure 10e, PIPs/cML MoS_2_ electrode exhibited superior sensitivity for the MMP-1 peptide compared to the non-imprinted counterpart (NIPs/cML MoS_2_) (Figure 10e). Similarly, another study employed the same peptide-imprinting strategy but incorporated MoS_2_ nanosheets into the polymer matrix, which amplified the electrochemical response and enabled the monitoring of CRISPR–dCas9-activated MMP-1 expression [126]. Therefore, by employing diverse electrode configurations in conjunction with peptide imprinting, high-sensitivity biosensing of MMP-1 could be successfully achieved. The unique combination of 2D materials and polymers enables complementary functionality in sensor platforms, achieving both enhanced sensing performance and device miniaturization, thereby paving the way for the development of next-generation sensor devices.

Complementary capabilities are also observed in flexible and wearable platforms. Zhao et al. [112] developed PANI and MXene nanocomposites for mechanically compliant sensing devices (Figure 10f). The hybrid coating on Au electrodes (Figure 10f) maintained stable electrical performance even when laminated onto a flexible PET substrate (Figure 10g) and subjected to various bending angles under gas detection environment (Figure 10h), highlighting its suitability for next-generation flexible and wearable electronics. Furthermore, the nanocomposite exhibited substantially higher sensitivity than pristine MXene across a wide concentration range (Figure 10i), confirming the synergistic interaction between the high surface area of MXene and the redox-active PANI matrix.

Collectively, these results underscore the multifunctionality of 2D–polymer hybrid architectures, demonstrating not only superior gas sensing and biosensing performance but also significant advantages in flexibility, durability, and device miniaturization, which are key attributes for future integrated sensing technologies.

### 4.2. Optoelectronics

Two-dimensional materials possess a large surface area and excellent optical properties, making them highly suitable as photosensor materials capable of detecting a broad spectral range from visible to ultraviolet light. When combined with polymers, interfacial characteristics such as electron transfer can be significantly enhanced, leading to the realization of 2D material–polymer heterostructures [4,127,128,129]. Although the following representative studies primarily employ small-molecule organic semiconductors rather than polymers, they offer important mechanistic insights into exciton dissociation, built-in-field engineering, and interfacial carrier transport that are directly transferable to 2D–polymer hybrid optoelectronic systems.

**Figure 11:**
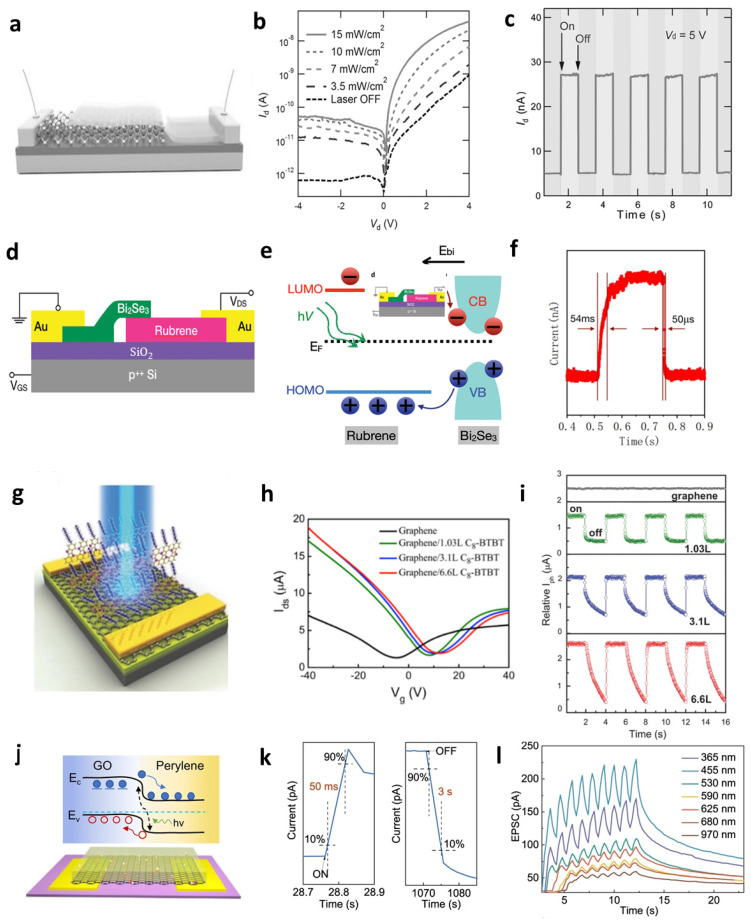
Optoelectronic characteristics of diverse 2D material–organic/polymer heterojunction photodetectors and photonic synapses. (**a**) Schematic illustration of the rubrene–MoS_2_ p–n heterojunction structure. (**b**) *I*_d_–*V*_d_ behavior under incremental optical excitation conditions. (**c**) Photocurrent switching behavior of the p–n junction device under periodic illumination. Reproduced with permission from Ref. [131] (Wiley-VCH, 2015). (**d**) Schematic of the rubrene–Bi_2_Se_3_ heterotransistor. (**e**) Energy band diagram of the rubrene–Bi_2_Se_3_ heterostructure. (**f**) Time-resolved photocurrent dynamics of the rubrene–Bi_2_Se_3_ heterojunction under periodic 532 nm light modulation. Reproduced with permission from Ref. [132] (Wiley-VCH, 2020). (**g**) Schematic of the C_8_-BTBT–graphene heterojunction phototransistor. (**h**) Evolution of the transfer characteristics of graphene FETs with increasing C_8_-BTBT layer thickness. (**i**) Dynamic photocurrent response of the devices measured under identical conditions (laser intensity: 7000 μW cm^−2^, *V*_ds_= 0.1 V, *V*_g_−*V*_0_ = 10 V). The top panel shows the photocurrent response of the pristine graphene device without C_8_-BTBT deposition. Reproduced with permission from Ref. [129] (Wiley-VCH, 2016). (**j**) Device layout and corresponding band diagram of the in-plane heterostructure. (**k**) Photocurrent rise and decay times. (**l**) EPSC characteristics triggered by nine consecutive optical pulses across UV, visible, and infrared wavelengths. Reproduced with permission from Ref. [133] (Springer Nature, 2022).

P–N heterojunction devices composed of 2D materials and polymer or small-molecule organic semiconductors have been widely reported [130]. Liu et al. [131] fabricated a MoS_2_–rubrene p–n diode (Figure 11a). While rubrene single crystals offer high carrier mobility, they inherently suffer from slow response times and persistent photoconductivity (PPC). These limitations can be effectively mitigated by forming heterostructures with 2D materials, which facilitate faster carrier separation and reduce trap-induced delays. The device was excited using a 532 nm laser capable of generating excitons in both MoS_2_ and rubrene. The photocurrent increased significantly with light intensity (Figure 11b), and the temporal photoresponse (Figure 11c) showed periodic switching synchronized with light ON–OFF cycles, confirming robust switching behavior under drain bias of 5 V. To address PPC, Pei et al. [132] proposed a rubrene–Bi_2_Se_3_ heterojunction phototransistor (Figure 11d). The device exhibited a significantly improved dynamic photoresponse, with a rise time of 54 ms and a decay time of 50 μs (Figure 11f). The rubrene–Bi_2_Se_3_ heterojunction achieved a faster photoresponse than pristine rubrene devices because the built-in electric field at the heterojunction quickly separates photogenerated carriers under illumination within rubrene’s bandgap, enabling rapid collection and current switching (Figure 11e).

Liu et al. [129] fabricated a planar C_8_-BTBT–graphene heterojunction phototransistor (Figure 11g). Bare graphene exhibited negligible photoresponse due to its low optical absorption (Figure 11i). By epitaxially growing the small-molecule organic semiconductor C_8_-BTBT on graphene, enhanced carrier mobility and interfacial electron transfer characteristics were achieved, with an interfacial charge-transfer efficiency (η_trans_) reaching approximately 41%. Furthermore, as the thickness of the C_8_-BTBT layer increased, a progressive positive shift in the charge neutrality point (CNP) of graphene was observed (Figure 11h), indicating improved interfacial electron transfer efficiency. Increasing the thickness of C_8_-BTBT enhanced both the photoresponsivity and response time under identical measurement conditions, with the photoresponsivity reaching up to R ≈ 4.76 × 10^5^ A·W^−1^ and the response time increasing to τ ≈ 830 ms. The improved photoresponsivity is due to increased light absorption in thicker C_8_-BTBT layers, while the longer response time results from extended carrier transport paths and trap-induced delays.

Zhang et al. [133] fabricated a high-quality, large-area perylene–GO heterobilayer. This structure forms a Type II band alignment arising from the p-type doping characteristics of GO, its defect-induced hole transport pathways, and the energy level offsets of perylene (Fermi level: 4.9 eV; HOMO: 6.1 eV) (Figure 11j). Upon illumination, electron–hole pairs are generated; the photogenerated electrons (e^−^) become trapped at defect sites in GO, while the holes (h^+^) migrate along the transport pathways, thereby producing a photoresponse current [134]. Time-resolved photoresponse analysis revealed a rise time (τr) of 50 ms and a fall time (τf) of 3 s (Figure 11k). The slow decay (τf) is attributed to persistent photocurrent caused by excess electrons trapped in GO defects. The fabricated detector demonstrates broadband optical perception and slow signal decay, enabling the emulation of visual perception and synaptic plasticity analogous to biological neurons [135]. Perylene exhibits strong absorption in the visible range, while GO, due to its defect-mediated absorption, enables broad spectral coverage from UV to NIR wavelengths. When a heterojunction is formed at the perylene–GO interface, the combined optical properties of both materials allow the device to respond to a wide spectral range from 365 to 970 nm. Under pulsed illumination across this broadband spectrum, the device exhibits a paired-pulse facilitation (PPF) behavior, in which excitatory postsynaptic currents (EPSCs) progressively accumulate (Figure 11i). This indicates that the heterostructure is not only an efficient optoelectronic sensor but can also be utilized in the memory field, including neuromorphic devices.

### 4.3. Neuromorphic Devices

Hybrid structures that integrate 2D materials with polymers enable highly versatile electronic and neuromorphic behaviors by combining the excellent electrical properties of atomically thin channels with the dielectric tunability, ionic mobility, and mechanical flexibility of polymer layers. These advantages allow for the implementation of non-volatile memory, low-power switching, and synaptic functions within a unified device architecture.

**Figure 12:**
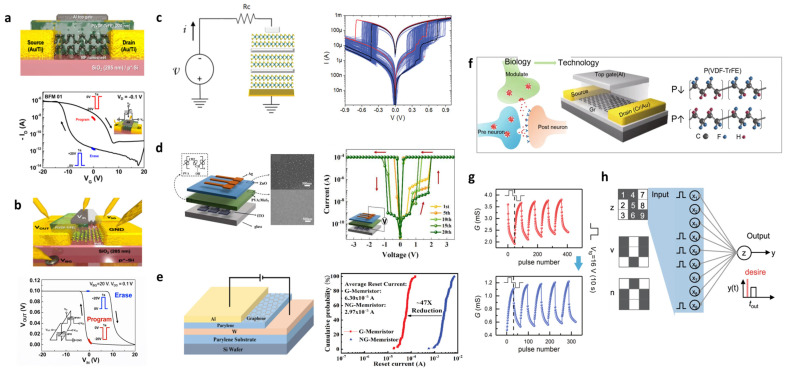
Electronic memory and neuromorphic characteristics of 2D material–polymer heterostructure devices. (**a**) BP-channel FeFET with a P(VDF–TrFE) ferroelectric gate dielectric, exhibiting a memory window of ~15 V and an ON–OFF ratio of ~10. (**b**) FeFET inverter in which ±20 V program/erase pulses shift the trip point, enabling multi-level logic-in-memory operation. Reproduced with permission from Ref. [26] (American Chemical Society, 2015). (**c**) MoS_2_/PMMA polymer heterostructure memristor showing bipolar resistive switching as evidenced by repeated *I*–*V* hysteresis loops. Reproduced with permission from Ref. [120] (American Chemical Society, 2020.) (**d**) ZnO–PVA:MoS_2_ bilayer memristor demonstrating stable bipolar switching and reproducible cycle-to-cycle endurance, confirmed by multiple I–V sweeps (1st–20th cycles). Reproduced with permission from Ref. [111] (MDPI, 2022). (**e**) Graphene–parylene memristor incorporating a graphene barrier layer that suppresses filament overgrowth, yielding an ~47-fold reduction in reset current and enabling low-power operation. Reproduced with permission from Ref. [123] (Wiley-VCH, 2019). (**f**) Graphene–P(VDF–TrFE) synaptic transistor in which ferroelectric polarization modulates graphene channel conductance, emulating biological potentiation and depression. (**g**) Analog conductance modulation under repeated positive and negative gate-pulse trains, reproducing long-term potentiation (LTP) and long-term depression (LTD) behaviors. (**h**) Neuromorphic pattern-recognition scheme using 3 × 3 spike-encoded inputs processed through complementary synapses to generate the desired output response. Reproduced with permission from Ref. [138] (Springer Nature, 2019).

First, these advantages are clearly demonstrated in ferroelectric field-effect transistors (FeFETs) [26,136,137]. Lee et al. [26] presented a BP-P(VDF–TrFE) top-gate FeFET structure, shown in Figure 12a, in which the P(VDF–TrFE) layer is deposited on few-layer BP flakes. Due to the strong C–F dipoles within the ferroelectric polymer, the n-type conduction of BP is effectively suppressed, resulting in a unipolar p-type memory transistor with enhanced ON–OFF characteristics. Well-defined program–erase states are observed when applying ±20 V gate pulses for 1 s, which generates a clear hysteresis window of ~15 V and an ON–OFF ratio of ~10^5^ (bottom figure of Figure 12a). The polarization-controlled threshold-voltage shift enables stable retention and high mobility, validating the effectiveness of ferroelectric gating in 2D–polymer hybrid transistors.

Furthermore, when the BP-FeFET is integrated into an inverter, non-volatile logic-in-memory can be directly implemented (Figure 12b). Program–erase pulses shift the trip point of the inverter, leading to two distinct V_OUT_ states. The resulting memory inverter demonstrates a memory window of ~13 V and a V_OUT_ memory efficiency of ~88%. Similarly, complementary FeCMOS circuits combining BP-FeFET (p-type) and MoS_2_-FeFET (n-type) achieve ~95% memory output efficiency and stable retention, confirming the feasibility of hybrid 2D–polymer ferroelectric devices for low-power neuromorphic computing.

In memristive devices, multiple 2D–polymer hybrid architectures have been reported. MoS_2_–PMMA hybrid structures show reproducible trap-mediated bipolar switching (Figure 12c) [120]. ZnO-protected PVA:MoS_2_ bilayers exhibit improved endurance (>10^3^ cycles) and retention (>3000 s), highlighting enhanced stability (Figure 12d) [111]. Additionally, in the graphene–parylene structure, the graphene barrier layer effectively suppressed filament growth, reducing the reset current by approximately 47 times, demonstrating that ultralow-power switching is possible (Figure 12e) [123]. In this structure, polymers play a key role in controlling dielectric properties, ion migration paths, and interface stabilization, while 2D materials provide thin, uniform conduction paths, enabling more precise switching [120,123].

These mechanisms can be extended to neuromorphic synaptic devices. Graphene–P(VDF–TrFE)-based synaptic transistors can mimic both synaptic strengthening and weakening by controlling conductivity depending on how the ferroelectric polarization changes (Figure 12f) [138]. When gate pulses are repeatedly sent, the conductance of the channel continuously changes, showing analog synaptic plasticity that reproduces long-term potentiation (LTP) and long-term depression (LTD) behaviors (Figure 12g). Additionally, when the elements are configured as an array, a pattern recognition function can be implemented by mapping a 3 × 3 input spike pattern to an output neuron (Figure 12h). However, it should be noted that ferroelectric polymers including P(VDF–TrFE) may exhibit thermal instability, susceptibility to UV exposure, and environmental sensitivity, which can influence long-term reliability and device uniformity. Addressing these challenges through material engineering, encapsulation strategies, and device-level optimization remains an important direction for the practical deployment of 2D–polymer hybrid-based neuromorphic devices.

### 4.4. Energy Devices

The field of energy devices has attracted considerable attention as a key area for next-generation electronic systems, including wearable, miniaturized, and high-capacity batteries. Current research primarily focuses on batteries and supercapacitors, and hybrid structures that combine 2D nanomaterials with polymers have demonstrated significant potential in enhancing both the electrochemical performance and mechanical stability of these devices. In particular, battery research is largely focused on improving electrolyte performance and developing solid-state electrolytes for Li-metal batteries [139].

**Figure 13:**
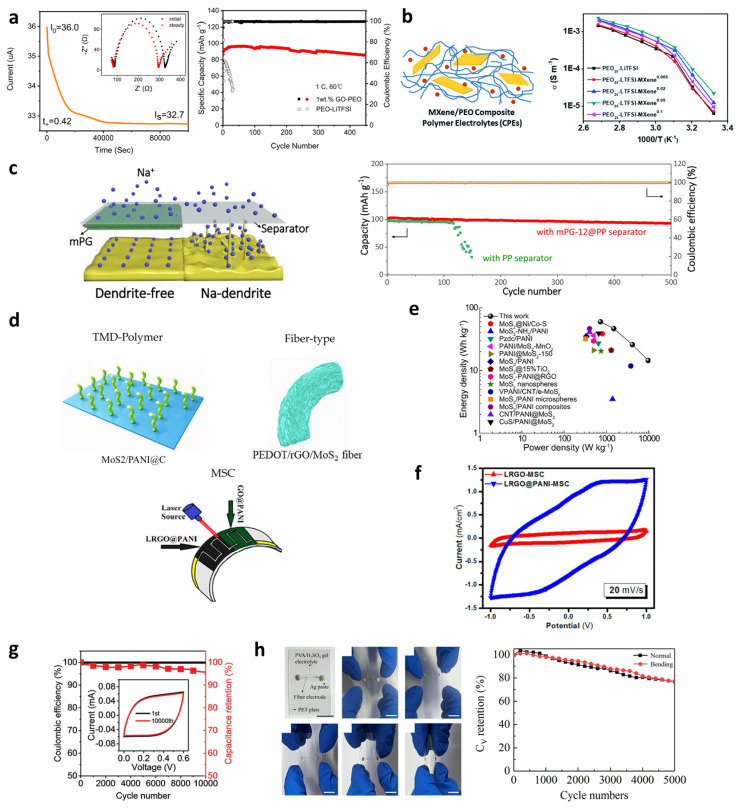
Applications and performance of 2D–polymer hybrid structures for emerging energy devices. (**a**) GO−PEO hybrid solid polymer electrolyte tLi^+^ current–time profile and cycling performance of the GO–PEO hybrid solid electrolyte. Reproduced with permission from Ref. [28] (American Chemical Society, 2021). (**b**) Structural diagram of the Ti_3_C_2_T_x_ MXene–PEO composite electrolyte and ionic conductivity variation with MXene content. Reproduced with permission from Ref. [140] (Royal Society of Chemistry, 2019). (**c**) Ion flow regulation via separator coating in a Na-metal battery and corresponding CE and specific capacity curves vs. cycle number. Reproduced with permission from Ref. [141] (Springer Nature, 2021). (**d**) Electrode structure schematics of TMD–polymer hybrid(MoS_2_/PANI@C), fiber-type(PEDOT/rGO/MoS_2_), and micro-supercapacitor (LRGO@PANI-MSC) devices. Reproduced with permission from Ref. [104] (American Chemical Society, 2023), and Ref. [105] (American Chemical Society, 2020). (**e**) Energy density curve of the MoS_2_–PANI electrode. Reproduced with permission from Ref. [117] (Wiley-VCH, 2023). (**f**) Comparative CV curves of LRGO-MSC and LRGO@PANI-MSC devices. Reproduced with permission from Ref. [105] (American Chemical Society, 2020). (**g**) Capacitance retention and Coulombic efficiency curves of PEDOT:PSS–MXene-based fiber-shaped supercapacitor. Reproduced with permission from Ref. [100] (Wiley-VCH, 2019). (**h**) Flexibility test setup and CV curves of PEDOT:PSS–rGO–MoS_2_-based supercapacitor during bent and unbent states. Reproduced with permission from Ref. [104] (American Chemical Society, 2023).

Wen et al. [28] utilized GO as a filler in a PEO-based solid polymer electrolyte. GO, with its oxygen-containing functional groups, disrupts the arrangement of PEO chains, suppressing crystallization and increasing the amorphous region. As Li^+^ ions migrate within the amorphous region along the segmental motion of polymer chains, this structural change directly leads to enhanced Li^+^ ion conductivity. In addition, the Li-ion transference number (tLi^+^) was markedly increased from 0.12 to 0.42, as the oxygen-containing functional groups on the GO surface (–COOH, –OH, epoxide) immobilized anions such as TFSI^−^. A higher tLi^+^ strengthens Li^+^-dominated conduction, suppresses dendrite growth, and contributes to the long-term cycling stability of the battery (98.7% retention after 450 cycles). Notably, the symmetric Li–Li cell exhibited a stable voltage profile for over 600 h at 0.1 mA cm^−2^, providing direct evidence of effective dendrite suppression (Figure 13a). A similar mechanism was reported using Ti_3_C_2_T_x_ MXene as a filler (Figure 13b) [103,140]. Ti_3_C_2_T_x_, a representative member of the 2D transition-metal carbide family, possesses a large specific surface area and hydrophilic surface terminations (O, OH, F). Pan et al. demonstrated that these characteristics, analogous to those of GO, enhance the interaction with PEO chains, suppress regular crystallization, increase the amorphous region, and thereby facilitate Li^+^ migration, ultimately improving ionic conductivity. Their incorporation of 3.6 wt% Ti_3_C_2_T_x_ into PEO achieved an ionic conductivity of 2.2 × 10^−5^ S cm^−1^ at 28 °C.

However, lithium has limitations such as scarcity and high cost. To overcome these issues, studies have focused on Na-metal electrodes, employing 2D nanomaterial–polymer hybrids to address the challenges of Na-metal batteries (Figure 13c). The use of Na-metal electrodes induces dendrite growth due to their high reactivity and unstable interfacial properties, thereby reducing battery stability and electrochemical efficiency. To solve this problem, Qin et al. [141] addressed the insufficient Na^+^ affinity of the conventional PP separator and improved it by applying a mesoporous (mPG) coating composed of polydopamine and multilayer graphene on its surface. This coating homogenizes Na^+^ ion flux through its porous structure and sodiophilic PDA surface groups, while the mechanical stability of the graphene layers suppresses dendrite penetration, thereby overcoming the limitations of the conventional separator. The graph on the right side of Figure 13c shows that the mPG-coated separator exhibited a Coulombic efficiency of ~99.8% and a capacity retention of ~90% over 500 cycles.

Supercapacitor research has primarily focused on enhancing energy density through electrode structure design, and Figure 13d shows representative electrode configurations designed using 2D nanomaterials and polymers, including three types of structures: a TMD–polymer configuration on the left side, a fiber-type structure on the right side, and a micro-supercapacitor (MSC) located at the bottom [104,105,116]. Dai et al. [117] employed MoS_2_@PANI electrodes, where the hydrogen bonding mechanism with NH_4_^+^ ions and sulfur vacancies enhanced electrical conductivity and storage performance. MoS_2_ facilitated NH_4_^+^ insertion and electron transport through its wide interlayer spacing and defect states generated by sulfur vacancies, while PANI provided hydrophilicity and conductivity to strengthen ion diffusion and charge transfer. This synergy enabled the device (Figure 13e) to achieve an energy density of 59.8 Wh kg^−1^ at a power density of 725 W kg^−1^, demonstrating superior performance compared with previous reports. In the study by Gholami et al. (Figure 13f), an interdigitated MSC employed laser-reduced GO electrodes incorporated with PANI. This electrode exhibited greatly enhanced charge transport through pseudocapacitance and electron delocalization pathways formed by π–π conjugations between PANI and graphene layers. As a result, ion diffusion and electron transfer were simultaneously promoted. In particular, compared with LRGO-MSC, LRGO@PANI-MSC exhibited a significantly increased current response in the CV curves with a high areal capacitance of 72 mF cm^2^, along with quasi-rectangular shapes and redox peaks, demonstrating the synergistic effects of EDLC and pseudocapacitance as well as the improvement in charge transport [105]. Zhang et al. [100] reported a coaxial fiber-shaped supercapacitor (FSC) in which PEDOT:PSS induced intersheet coupling and the alignment of MXene sheets, thereby refining electron transport pathways and improving conductivity to a record high of ≈1489 S cm^−1^ (Figure 13g). This device maintained ≈95% of its capacitance and ≈100% Coulombic efficiency after 10,000 cycles, confirming its long-term cycling stability. Furthermore, the device retained 96% of its capacitance even under repeated 100% stretching, verifying its structural integrity against mechanical stress. Figure 13h presents a flexible fiber-shaped supercapacitor (FSSC) based on PEDOT:PSS–rGO–MoS_2_. Zhou et al. demonstrated that the synergistic effects of MoS_2_ pseudocapacitance, rGO mechanical support and ion diffusion, and PEDOT:PSS conductivity and flexibility enabled the device to deliver a high volumetric capacitance of 325.8 F cm^−3^ and an energy density of 6.9 mWh cm^−3^. Moreover, it retained nearly its entire capacitance even after 1000 bending cycles, proving its high durability and applicability to flexible electronic devices.

## 5. Conclusions

Two-dimensional materials and polymers together form a highly versatile class of hybrid interfaces that unlock functionalities unattainable by either component alone. As summarized throughout this review, the structural configurations ranging from embedded nanocomposites and stacked heterostructures to covalently functionalized surfaces and fiber–network hybrids offer tunable pathways for electronic interaction, mechanical reinforcement, and chemical modulation. Advances in fabrication strategies, including solution processing, surface-directed polymerization, and vapor-phase or interfacial deposition, now enable precise control over interface chemistry and device-level integration while preserving the intrinsic properties of 2D materials. These synergistic hybrid systems have already demonstrated their impact across sensors, optoelectronics, memory and neuromorphic platforms, and energy devices, where interfacial engineering plays a central role in achieving high performance, stability, and multifunctionality. Despite these impressive benchmarks, the field continues to struggle with the lack of standardized characterization protocols and universal design rules, which often limits the reproducibility and large-scale integration of these hybrid systems. Looking ahead, key opportunities lie in scalable manufacturing, long-term reliability, deterministic interface design, and the development of hybrid architectures tailored for heterogeneous integration and flexible, wearable, or bio-interactive electronics. Continued advances at the intersection of polymer chemistry and 2D material science are expected to drive the next generation of multifunctional, reconfigurable, and application-adaptive electronic and energy systems.

## Data Availability

No new data were created or analyzed in this study. Data sharing is not applicable to this article.

## Abbreviations

The following abbreviations are used in this manuscript:

PMMA	Poly(methyl methacrylate)
P(VDF–TrFE)	Poly(vinylidene fluoride trifluorethylene)
SEM	Scanning electron microscopic
WS_2_	Tungsten disulfide
PVOH	Polyvinyl alcohol
XRD	X-ray diffraction
DFT	Density functional theory
HOMO	Highest occupied molecular orbital
LUMO	Lowest unoccupied molecular orbital
GO	Graphene oxide
P(VDF–TrFE–CFE)	Poly(vinylidene fluoride-trifluoroethylene-chlorofluoroethylene)
PVP	polyvinylpyrrolidone
PL	Photoluminescence
PEI	Polyethyleneimine
MoS_2_	Molybdenum disulfide
XPS	X-ray photoelectron spectroscopy
PVA	Polyvinyl alcohol
PECVD	Plasma-enhanced chemical vapor deposition
ALD	Atomic layer deposition
CVD	Chemical vapor deposition
PEO	Poly(ethylene oxide)
DMF	Dimethylformamide
LiTFSI	Lithium bis(trifluoromethanesulfonyl)imide
ReS_2_	Rhenium disulfide
TEM	Transmission electron microscope
PANI	Polyaniline
UPS	Ultraviolet photoelectron spectroscopy
UV	Ultraviolet
NIR	Near-Infrared
PP	Polypropylene
BP	Black phosphorus
